# Correlation of Thyroid Transcription Factor-1 Expression with *EGFR* Mutations in Non-Small-Cell Lung Cancer: A Meta-Analysis

**DOI:** 10.3390/medicina55020041

**Published:** 2019-02-07

**Authors:** Hyeong Su Kim, Jung Han Kim, Boram Han, Dae Ro Choi

**Affiliations:** 1 Division of Hemato-Oncology, Department of Internal Medicine, Kangnam Sacred-Heart Hospital, Hallym University Medical Center, Hallym University College of Medicine, Seoul 07441, Korea; 2 Division of Hemato-Oncology, Department of Internal Medicine, Hallym University Medical Center, Anyang 14068, Korea; borbor@hallym.or.kr (B.H.); choidaero@hallym.or.kr (D.R.C.)

**Keywords:** thyroid transcription factor-1, *EGFR* mutation, non-small-cell lung cancer, biomarker, meta-analysis

## Abstract

*Objectives:* This meta-analysis investigated the relationship between thyroid transcription factor-1 (TTF-1) expression and epidermal growth factor receptor (*EGFR*) mutations in non-small-cell lung cancer (NSCLC) to clarify whether TTF-1 can be a potential surrogate marker for *EGFR* mutation status in advanced NSLCL. *Methods:* A systematic searching of databases, including PubMed, EMBASE, Cochrane Library, and Google Scholar, was performed to identify studies assessing the correlation of TTF-1 expression with EGFR mutations. From 17 studies, 9764 patients were included in the combined analysis of odds ratio (OR) for the correlation between TTF-1 expression and *EGFR* mutations. *Results:* Compared with NSCLCs showing negative TTF-1 expression, tumors harboring TTF-1 overexpression showed a significantly higher rate of *EGFR* mutations (OR = 5.19, 95% confidence interval: 3.60–7.47, *p* < 0.00001). This correlation was observed in both subgroups of East Asian (OR = 4.33, 95% CI: 3.46–5.41, *p* < 0.00001) and European patients (OR = 4.64, 95% CI: 1.41–15.28, *p* < 0.01). In addition, TTF-1 expression was significantly associated with *EGFR* mutations in exon 19 (OR = 4.63, 95% CI: 2.89–7.41, *p* < 0.00001) as well as exon 21 (OR = 3.16, 95% CI: 1.04–9.60, *p* = 0.04). *Conclusions:* This meta-analysis demonstrates a significant correlation between TTF-1 expression and *EGFR* mutations in patients with NSCLC. The status of TTF-1 expression may be a biomarker to guide anticancer treatment in patients with NSCLC and unknown *EGFR* mutation status.

## 1. Introduction

Lung cancer is the second most common malignancy in both genders worldwide [1]. It still remains the leading cause of cancer-associated deaths [1,2], although systemic chemotherapy or immune checkpoint inhibitors can significantly improve prognosis for patients with advanced non-small-cell lung cancer (NSCLC) [3,4,5]. For patients with epidermal growth factor receptor (*EGFR*)-mutant NSCLC, targeted therapy with tyrosine kinase inhibitors (TKIs) can significantly prolong survival [6,7].

Thyroid transcription factor-1 (TTF-1) is a regulatory transcription factor for tissue-specific genes [8]. It is expressed in the thyroid, forebrain, or lungs, playing a physiologic role during their development. In the normal lungs, TTF-1 helps to maintain functions of terminal respiratory unit cells [9]. High TTF-1 expression by immunohistochemistry (IHC) has been observed in 70–90% of primary lung adenocarcinomas (ADCs), while almost all squamous cell carcinomas are negative for TTF-1 IHC. Therefore, it has been considered a specific marker of ADCs of the lung. In addition, TTF-1 overexpression is a favorable prognostic factor not only in early-stage but also in advanced non-squamous NSCLC [10,11].

EGFR is a member of the hermaphrodite (HER) family of tyrosine kinase receptors, and somatic activating mutations in the adenosine triphosphate (ATP)-binding site of EGFR result in a more effective binding of EGFR TKIs [12,13]. *EGFR* gene mutations in four kinase domains (exons 18–21) comprise in-frame deletions, in-frame insertions/duplications, and point mutations [14,15]. NSCLC patients with *EGFR* mutations can achieve better progression-free survival and overall survival when treated with an EGFR TKI as first-line treatment rather than chemotherapy [6,16,17,18]. Therefore, it is essential to determine the *EGFR* mutation status of patients with advanced NSCLC when planning anticancer therapy.

However, for some patients, it is not easy to determine the *EGFR* mutation status because of inadequate tumor specimen or expense. Therefore, the identification of other pathologic markers that can predict *EGFR* mutation status may be very useful in clinical practice. In NSCLC, both TTF-1 expression and *EGFR* mutations are closely related to the female gender, non-smoking status, and ADC [13,19,20,21,22]. In addition, some studies suggested that TTF-1 expression had a significant positive correlation with *EGFR* mutations [21,22]. This meta-analysis assessed the relationship between TTF-1 expression and *EGFR* mutations in NSCLC to clarify whether TTF-1 can be a potential predictive biomarker for *EGFR* mutation status in patients with NSLCL.

## 2. Materials and Methods

### 2.1. Publication Search Strategy

This meta-analysis was done according to the Preferred Reporting Items for Systematic Reviews and Meta-Analyses (PRISMA) guidelines [23]. A systematic search of the databases including PubMed, EMBASE, Cochrane Library, and Google Scholar (up to December 2018) was performed to identify studies assessing the correlation of TTF-1 expression with *EGFR* mutations. The search used a combination of the following terms: “epidermal growth factor receptor” or “EGFR” AND “mutation” AND “thyroid transcription factor-1” or “TTF-1” AND “non-small-cell lung cancer” or “NSCLC” or “lung cancer.” All of the relevant articles identified by the related article function were also included in the analysis. The references reported in the identified articles were also reviewed to complete the search process.

### 2.2. Eligibility Criteria

Eligible studies should meet the following inclusion criteria: (i) patients with pathologically confirmed NSCLC; (ii) analysis of *EGFR* mutations in exons 19 and 21; (iii) IHC test for TTF-1 expression in lung cancer tissue; (iv) the use of adequate IHC methods and criteria for positive TTF-1 staining; and (v) prospective or retrospective cohort studies assessing the correlation of TTF-1 expression with *EGFR* mutations.

### 2.3. Article Review and Data Extraction

Two authors (D.R.C. and B.H.) independently searched the databases and extracted data from the selected studies. The following data were extracted from each study: the first author, year of publication, study design, inclusion period, country, sample size, histology, disease stage, TTF-1 expression status, IHC criteria for positive expression, *EGFR* mutation status, and detecting method.

### 2.4. Quality Assessment

The methodological quality of included studies was scored based on the Newcastle–Ottawa System (NOS) with the score range of zero to nine [24]. Studies having a score ≥ six were considered to have a high quality.

### 2.5. Statistical Analysis

The strength of the association between TTF-1 expression and *EGFR* mutations was shown as odds ratios (ORs) with 95% confidence intervals (CIs). If the study did not report the OR or 95% CI directly, we calculated them from available data by using the Engauge Digitizer software. The heterogeneity of the individual ORs was estimated using the chi-squared test with significance being set at *p <* 0.1. The total variation among studies was estimated by anI^2^ inconsistency test, where I^2^ > 50% was considered to indicate significant heterogeneity. If there was heterogeneity among studies, we used the random effect model based on the DerSimonian–Laird method to pool the OR. Otherwise (*p* ≥ 0.1 and I^2^ ≤ 50%), the fixed effect model based on the Mantel–Haenszel method was selected. Subgroup analyses were planned according to the ethnicity and mutational types. The sensitivity analysis was performed to detect the influence of individual trials on the pooled results by removing one trial each time. Forest plots were produced to show a summary estimate of the combined results of all the studies. Each square represented the OR point estimate, and its size was proportional to the weight of the study. The location of the diamond represented the estimated effect size, and its width reflected the precision of the estimate.

The potential publication bias was assessed by visual inspection of the funnel plot [25]. For quantitative analyses, Egger’s test and Begg’s tests were performed using the statistical software packages R [26]. Statistical significances were considered when a *p*-value was less than 0.05.

## 3. Results

### 3.1. Search Results

**Figure 1:**
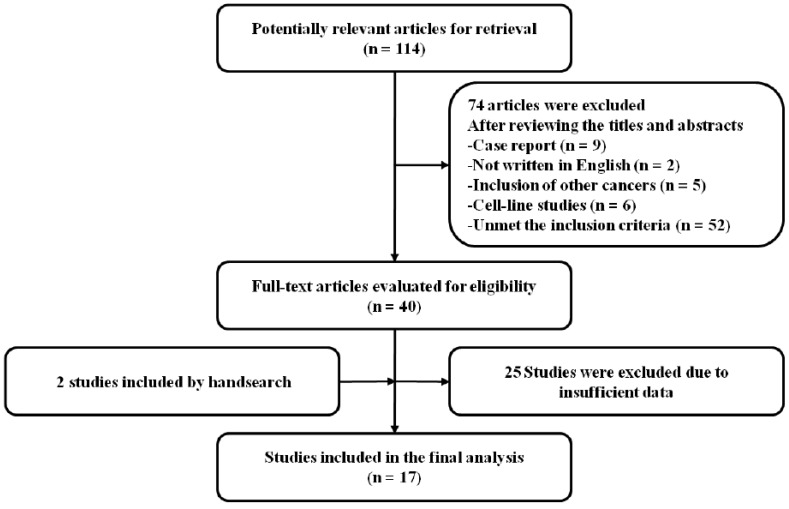
Flow diagram of search process.

The search process identified 114 potentially relevant articles; however, 74 articles were excluded by screening of the titles and abstracts. Of the remaining 40 studies, 25 articles that failed to meet the inclusion criteria were further excluded. We manually searched the reference lists of the selected articles and found two more relevant articles. Eventually, a total of 17 studies were included in the meta-analysis [21,27,28,29,30,31,32,33,34,35,36,37,38,39,40,41,42] (Figure 1).

### 3.2. Characteristics of the Included Studies

The main characteristics of the studies selected for this meta-analysis are summarized in Table 1. Except for two studies without any description about study design [35,40], seven recruited patients prospectively [21,27,30,32,34,39,41], and the remaining studies were performed retrospectively [28,29,31,33,36,37,38,42]. The NOS scores were more than seven in all of the included studies, suggesting a good methodological quality.

The criteria for the positivity of TTF-1 expression in IHC staining varied across studies; however, the rates of TTF-1 expression in most of the included studies were 80–90%. Six studies contained NSCLC [28,29,31,32,36,41], and 10 studies only included ADC [21,27,30,33,34,35,38,39,40,42]. Two studies included patients with stages I–III ADC [21,38], and five were conducted in patients with advanced disease (stages III–IV) [30,32,36,37,42]. 

The *EGFR* mutations were usually detected by polymerase chain reaction (PCR) and direct DNA sequencing methods, or by amplification refractory mutation system (ARMS) analysis. Most of the studies examined four kinase domains in exons 18–21; however, some screened for *EGFR* mutations in only exons 19 and 21 [21,31,36].

### 3.3. Correlation of TTF-1 Expression and EGFR Mutations

#### 3.3.1. Overall

**Figure 2:**
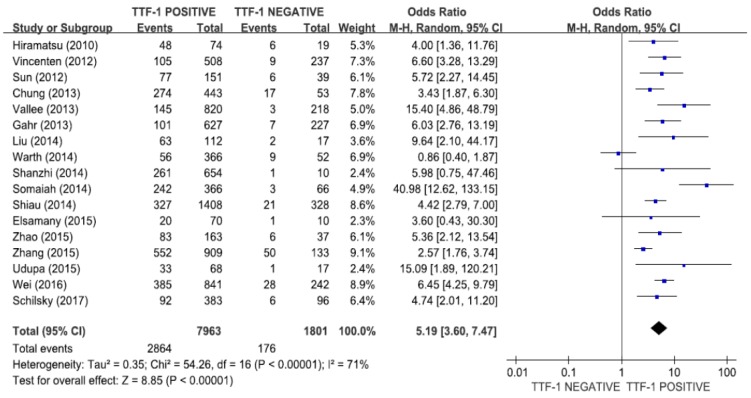
Forest plot for the correlation between TTF-1 expression and *EGFR* mutations in NSCLC.

A total of 9764 patients from the 17 studies were included in the meta-analysis to determine the association of TTF-1 expression and *EGFR* mutations [21,27,28,29,30,31,32,33,34,35,36,37,38,39,40,41,42]. The combined OR of 5.19 (95% CI: 3.60–7.47, *p* < 0.00001, random-effects model, Figure 2) indicated that NSCLCs with TTF-1 overexpression exhibited significantly higher rate of *EGFR* mutations. There was a significant heterogeneity among studies (X^2^ = 54.26, *p* < 0.00001, I^2^ = 71%).

#### 3.3.2. Subgroup Analysis According to the Ethnic Group

**Figure 3:**
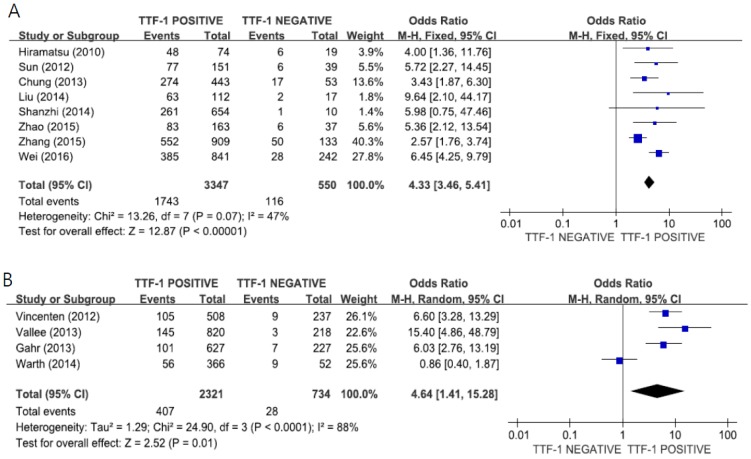
Forest plots for the correlation of TTF-1 expression and *EGFR* mutations according to the ethnicity: East Asian (**A**) and European (**B**).

When we performed the subgroup analysis according to the region, the combined ORs were 4.33 (95% CI: 3.46–5.41, *p* < 0.00001, fixed-effects model) for patients in East Asia (Figure 3A) and 4.64 (95% CI: 1.41–15.28, *p* < 0.01, random-effects model) for patients in Europe (Figure 3B).

#### 3.3.3. Subgroup Analysis According to the Gender

**Figure 4:**
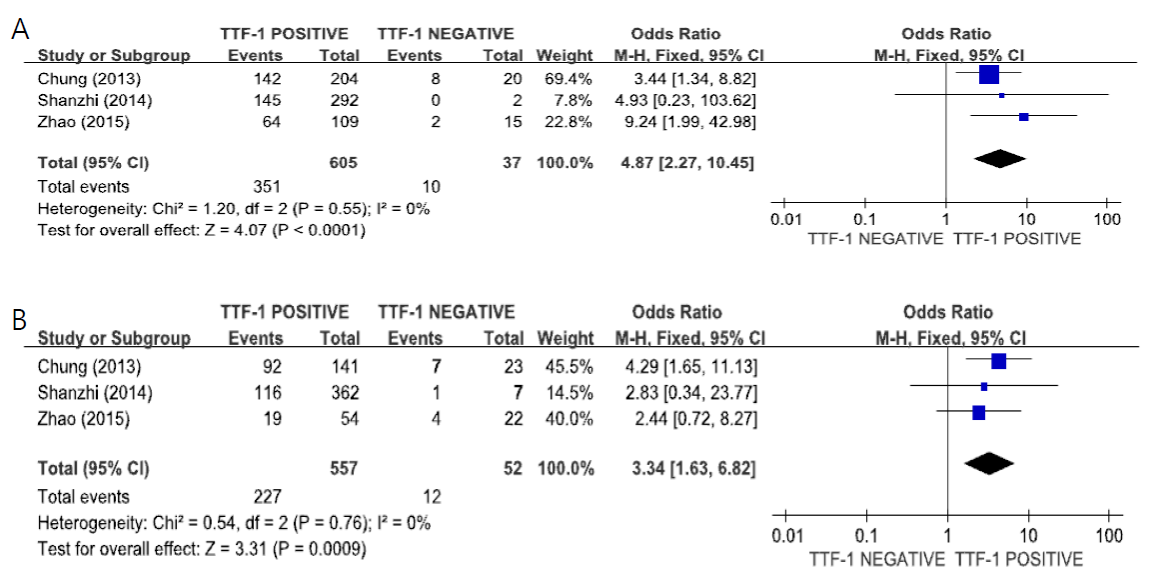
Forest plots for the correlation of TTF-1 expression and *EGFR* mutations according to the gender: female (**A**) and male (**B**).

The subgroup analysis using data from three studies [30,34,38] indicated there was a significant correlation between TTF-1 expression and *EGFR* mutations in both female (OR = 4.87, 95%CI 2.27–10.45, fixed-effect model, Figure 4A, *p* < 0.0001) and male patients (OR = 3.34, 95%CI 1.43–6.82, fixed-effect model, Figure 4B, *p* = 0.0009).

#### 3.3.4. Subgroup Analysis According to the Mutational Subtype

**Figure 5:**
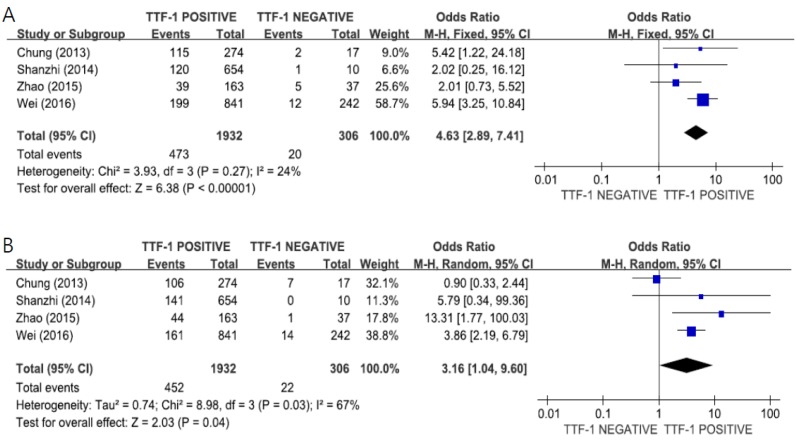
Forest plots for the correlation of TTF-1 expression and *EGFR* mutation according to the mutational subtype: exon 19 (**A**) and exon 21 (**B**).

The pooled data from four studies [30,34,38,41] revealed a significant correlation between TTF-1 expression and *EGFR* mutations in both exon 19 (OR = 4.63, 95% CI: 2.89–7.41, *p* < 0.00001, fixed-effect model, Figure 5A) and exon 21 (OR = 3.16, 95% CI: 1.04–9.60, *p* = 0.04, random-effects model, Figure 5B).

### 3.4. Publication Bias

**Figure 6:**
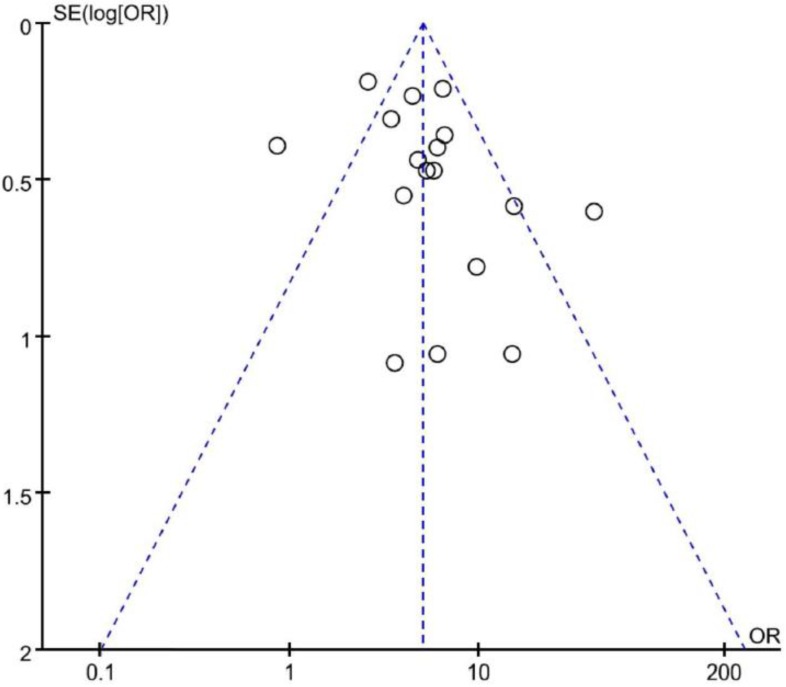
Funnel plot for publication bias.

Visual inspection of the funnel plot for ORs showed symmetry, indicating that there was no substantial publication bias (Figure 6). Egger’s and Begg’s tests also indicated no evidence of substantial publication bias, with *p*-values of 0.172 and 0.152, respectively.

## 4. Discussion

Over the past two decades, several major progresses have been made toward the personalized treatment for patients with NSCLC. The first breakthrough was the discovery of *EGFR* mutations. TKIs are small molecular agents targeting *EGFR* mutations that have revolutionized the treatment of NSCLC, leading to improved survival in patients with advanced or metastatic *EGFR*-mutant tumor [6,7]. Therefore, it is essential to screen for *EGFR* mutations before introducing anticancer treatment for patients with advanced NSCLC.

Studies evaluating the prognostic value of TTF-1 expression in NSCLC reported TTF-1 expression to be an independent predictor of survival [10,11,43,44]. In the North East Japan 002 study, investigators reported that the rate of *EGFR* mutations was higher in patients with ADC positive for TTF-1 expression [6]. In particular, Asians, women, and non-smokers revealed a higher rate of *EGFR* mutations and TTF-1 positive expression. Based on these findings, some researchers hypothesized that TTF-1 expression is significantly correlated with *EGFR* mutations in patients with lung ADC [34].

In the current meta-analysis, we combined the data from 17 studies, including 9764 patients, to determine whether positive TTF-1 expression by IHC can be a surrogate marker for *EGFR* mutation status in NSCLC. The results revealed that *EGFR* mutations were significantly correlated with TTF-1 overexpression in patients with NSCLC (OR = 5.34, 95% CI: 3.54–8.04, *p* < 0.00001). Both high TTF-1 expression and *EGFR* mutations have been known to be significantly associated with Asian, women, and non-smokers. However, our subgroup analysis according to the ethnicity indicated that the correlation of TTF-1 expression and *EGFR* mutations was observed in European patients (OR = 4.64, 95% CI: 1.41–15.28, *p* < 0.01) as well as East Asian (OR = 4.33, 95% CI: 3.46–5.41, *p* < 0.00001). In addition, in the subgroup analysis according to the gender, the significant correlation between TTF-1 expression and *EGFR* mutations was observed not only in female (OR = 4.87, 95% CI 2.27–10.45, *p* < 0.0001), but also in male patients with NSCLC (OR = 3.34, 95% CI 1.43–6.82, *p* = 0.0009).

Among *EGFR* mutations, deletion in exon 19 and L858R mutation in exon 21 are common, accounting for more than 90% of all *EGFR* mutations. Some studies conducted subgroup analysis according to *EGFR* mutation subtype and reported that positive TTF-1 expression was only significantly correlated with *EGFR* mutation in exon 21, and not on exon 19 [34,38]. However, Zhao et al. also observed a significant correlation of TTF-1 positivity with *EGFR* mutation in exon 21 in Chinese patients with lung ADC [38]. In the current meta-analysis, the pooled data from four studies [30,34,38,41] indicated a significant relationship between TTF-1 expression and *EGFR* mutation in both exon 19 (OR = 4.63, 95% CI: 2.89–7.41, *p* < 0.00001) and exon 21 (OR = 3.16, 95% CI: 1.04–9.60, *p* = 0.04).

Several studies reported a high negative predictive value (88.2–97%) of TTF-1 for the presence of activating *EGFR* gene mutations [21,28,35,40]. In this study, only 9.8% (176 of 1801) of TTF-1–negative NSCLC cases had *EGFR* mutations. Overall, the negative predictive value of TTF-1 for *EGFR* mutations was 90.2%. However, when including only patients in Europe, where the rate of *EGFR* mutations is low, the negative predictive value of TTF-1 increased to 96.2%. Therefore, when the status of *EGFR* mutations cannot be tested in a timely manner because of inadequate tumor tissue or when treatment for NSCLC is urgent for clinical reasons, the IHC staining result of TTF-1 may be used to guide systemic anticancer therapy. In particular, negative TTF-1 expression can be a surrogate marker to recommend conventional chemotherapy.

This study has several limitations. First, the included studies showed considerable diversity in the methods used for TTF-1 staining and *EGFR* mutation detection. In addition, the cut-off criteria for positive TTF-1 staining also varied among studies. Second, the rate of TTF-1 overexpression and *EGFR* mutations are significantly higher with NSCLC patients with no smoking history. However, we could not perform a subgroup analysis according to the smoking status because of the limited data. Third, the substantial heterogeneity observed among studies could not be interpreted thoroughly, even though the random-effects model was selected. Finally, about half of the included studies were retrospective and therefore may carry the biases inherent to the study design.

## 5. Conclusions

This meta-analysis indicates a significant correlation between TTF-1 overexpression and *EGFR* mutations status in patients with NSCLC. In clinical practice, the status of TTF-1 expression may be a biomarker to guide anticancer therapy in advanced NSCLC patients with unknown *EGFR* mutation status. Especially, negative TTF-1 expression has such a high negative predictive value for *EGFR* mutations that it might be a surrogate marker to recommend conventional chemotherapy first when the status of *EGFR* mutations cannot be tested in a timely manner.

## Figures and Tables

**Table 1:** Summary of the 17 included studies.

First Author (Year) [Ref.]	Country	Design	Sample Size	Inclusion Period	Pathology	Tumor Stage	IHC Criteria for TTF-1 (+): Nuclear Staining	Test for EGFR Mutations	TTF-1 Expression	*EGFR* MT a/t TTF-1 (%)	Significance	NOS Score
Hiramatsu (2010) [27]	Japan	Pro	93	1998–2001	ADC	I–IV	Stronger than xenograft staining	PCR	(+): 74 (79.6%) (−): 19 (20.4%)	48 (64.9%) 6 (31.6%)	*p* = 0.017	6
Vincenten (2012) [28]	Netherlands	Retro	745	2004–2010	NSCLC	I–IV	Percentage (0–100%) × intensity (0–3) ≥10	PCR sequencing	(+): 508 (68.2%) (−): 237 (31.8%)	105 (20.7%) 9 (3.8%)	*p* < 0.00001	8
Sun (2012) [29]	Korea	Retro	190	2006–2010	NSCLC	NA	Percentage (0–100%) × intensity (0–3) >100	PCR sequencing	(+): 151 (79.4%) (−): 39 (20.6%)	77 (60.0%) 6 (15.4%)	*p* < 0.001	8
Chung (2012) [30]	Taiwan	Pro	496	2004–2009	ADC	IIIB–IV	Any definite nuclear staining	PCR sequencing	(+): 443 (89.3%) (−): 53 (10.7%)	274 (61.9%) 17 (32.1%)	*p* < 0.001	8
Vallee (2013) [31]	France	Retro	1038	2010–2012	NSCLC	I–IV	NA	PCR	(+): 820 (79.0%) (−): 218 (21.0%	145 (17.7%) 3 (1.4%)	*p* < 0.0001	8
Gahr (2013) [32]	Germany	Pro	854	2010	NSCLC	Mainly IV	NA	Sanger sequencing	(+): 627 (73.4%) (−): 227 (26.6%)	101 (16.1%) 7 (3.1%)	*p* < 0.001	8
Liu (2014) [21]	China	Pro	139	2008–2011	ADC	I–III	Intensity (0–3) x reactivity (0–100) > 100	ARMS PCR	(+): 122 (87.8%) (−): 17 (12.2%)	63 (51.6%) 2 (11.8%)	*p* = 0.002	7
Warth (2014) [33]	Germany	Retro	418	2002–2008	ADC	I–IV	NA	Sanger sequencing	(+): 366 (87.6%) (−): 52 (12.4%)	56 (15.3%) 9 (17.3%)	*p* = 0.685	8
Shanzhi (2014) [34]	China	Pro	664	2010–2013	ADC	I–IV	>10% of tumor cells	PCR sequencing	(+): 654 (98.5%) (−): 10 (1.5%)	261 (39.9%) 1 (10%)	*p* < 0.001	8
Somaiah (2014) [35]	USA	NA	431	NA	ADC	NA	NA	Allele-specific PCR	(+): 366 (84.9%) (−): 66 (15.3%)	242 (66.1%) 3 (4.5%)	*p* < 0.00001	8
Shiau (2014) [36]	Canada	Retro	1736	2010–2012	Non-SQCC	Mainly III–IV	NA	PCR	(+): 1408 (81.1) (−): 328 (18.9)	327 (23.2) 21 (6.4)	*p* < 0.00001	8
Elsamany (2015) [37]	Egypt	Retro	80	2011–2012	Non-SQCC	IIIB–IV	NA	NA	(+): 70 ((87.5%) (−): 10 (12.5%)	20 (28.6%) 1 (10%)	*p* = 0.28	6
Zhao (2015) [38]	Taiwan	Retro	200	2008–2013	ADC	I–IV	≥ 10% of tumor cells	EGFR liquid chip	(+): 163 (81.5%) (−): 37 (18.5%)	83 (50.9%) 6 (16.2%)	*p* = 0.000	7
Zhang (2015) [39]	China	Pro	1042	2008–2013	ADC	I–III	Any positive nuclear staining	PCR	(+): 909 (87.26%) (−): 133 (12.8%)	552 (60.7%) 50 (37.6%)	*p* < 0.001	8
Udupa (2015) [40]	India	NA	85	2009-2013	ADC	I–IV	NA	ARMS real-time PCR	(+): 68 (80%) (−): 17 (20%)	33 (48.5%) 1 (5.9%)	*p* < 0.00001	6
Wei (2016) [41]	China	Pro	1083	2010–2016	NSCLC	I–IV	Tan or brown nuclear staining	ARMS PCR	(+): 841 (77.7%) (−): 242 (22.3%)	385 (45.8%) 28 (11.6%)	*p* < 0.001	8
Schilsky (2017) [42]	USA	Retro	479	2009–2011	ADC	IV	Any nuclear reactivity	NA	(+): 383 (80.0%) (−): 96 (20.0%)	92 (24.0%) 6 (6.3%)	*p* < 0.001	8

EGFR, epidermal growth factor receptor; NOS, Newcastle–Ottawa System; NSCLC, non-small-cell lung cancer; Pro, prospective; Retro, retrospective; pts, Patients; ADC, adenocarcinoma; SQCC, squamous cell carcinoma; TTF-1, thyroid transcription factor 1; ARMS, amplification refractory mutation system; PCR, polymerase chain reaction; IHC, immunohistochemistry; a/t, according to; NA, not available.
